# Structural Characterization and In Vitro Antioxidant Activity of a Novel Polysaccharide from Summer–Autumn Tea

**DOI:** 10.3390/foods13060821

**Published:** 2024-03-07

**Authors:** Miao Cao, Zheng Cao, Juanjuan Tian, Wenping Lv, Hongxin Wang

**Affiliations:** 1 Department of Tea and Food Science, Jiangsu Vocational College of Agriculture and Forestry, Zhenjiang 212400, China; michaelmcao@163.com (M.C.);; 2 School of Food Science & Technology, Jiangnan University, Wuxi 214122, China; 3 The State Key Laboratory of Food Science & Technology, Jiangnan University, Wuxi 214122, China

**Keywords:** summer–autumn tea, polysaccharide, structural characterization, antioxidant activity

## Abstract

To enhance the utilization of summer–autumn tea, a water-soluble polysaccharide (D1N1) was isolated through a series of techniques including hot water extraction, ethanol precipitation, and column chromatography. The structure of D1N1 was determined through the utilization of ultraviolet, Fourier-transform infrared, high-performance anion-exchange chromatography, gas chromatography–tandem mass spectrometry, and nuclear magnetic resonance. The results revealed that glucose was the predominant component of D1N1, accounting for 95% of its composition. Additionally, D1N1 also contained galactose, arabinose, and rhamnose. The molecular weight (Mw) of D1N1 was determined to be 224.71 kDa. The backbone of D1N1 consisted of →4)-α-D -Glc*p* (1→, →3,4)-α-D-Gal*p*-(1→, →4,6)-α-D -Glc*p* (1→ at a molar ratio of 35:1:1, and branching at the O-3 position of →3,4)-α-D-Gal*p*-(1→ and O-6 position of →4,6)-α-D-Glc*p* (1→ with α-D -Glc*p* (1→. In addition, the antioxidant activity of D1N1 was also evaluated. D1N1 exhibited excellent antioxidant bioactivity against the DPPH, superoxide anion radical, and ABTS^+^ radical. These findings provide a theoretical basis for the application of summer–autumn tea polysaccharide as a potential functional food.

## 1. Introduction

Tea is a widely consumed beverage derived from the dried leaves and buds of plants. It has been consumed in Asia for 5000 years, especially in China, India, and Thailand [1]. Tea is favored by the majority of people because of the presence of numerous bioactive compounds, including tea polyphenols, tea polysaccharides (TPSs), tea pigments, alkaloids, amino acids, proteins, and other advantageous substances [2]. Most studies have focused on the bioactive effects of tea polyphenols, which are small-molecule compounds [3]. Tea polysaccharides, a major class of chemicals in tea, have attracted increasing attention from researchers since the late 1980s [4]. TPSs have drawn less attention due to their complex structures. The majority of TPSs are composed of 2–10 monosaccharides, including glucose (Glu), rhamnose (Rha), arabinose (Ara), mannose (Man), ribose (Rib), xylose (Xyl), galactose (Gal), fucose (Fuc), galacturonic acid (GalA), and glucuronic acid (GluA) [5,6]. In addition, they possess a wide range of molecular weights, varying from 0.472 to 3140 kDa [7]. Moreover, the main glycosidic bond types of TPSs are diverse, and the elucidation of the advanced structures and conformations of TPSs also faces enormous challenges due to limited research results. For instance, the molecular chain conformation of TPSs in solution is typically described as either spherical, random coil, or helical in shape. Therefore, the structure of polysaccharides is highly complex.

TPSs have been observed to possess a diverse range of biological functionalities, including antioxidant, anti-tumor, anti-obesity, and immunity-enhancing properties [4,7,8]. With the development of modern science and technology, it has been gradually discovered that the biological activities of polysaccharides are closely related to their structures, including chemical structures (monosaccharide composition, linear and branch structures), molecular weights, and chain conformations [9,10]. Some studies have reported that TPSs with a larger molecular weight (Mw) had better antioxidant activity than those with a smaller Mw [11,12]. Meanwhile, the ability to scavenge 2,2-diphenyl-1-picrylhydrazyl (DPPH) radicals was observed in relation to the presence of glucose linked at position 1→6 and arabinose linked at position 1→4 [13]. Therefore, due to the structural diversity and complexity of polysaccharides, the structure–activity relationship between polysaccharides and their activities is still unclear and needs to be further investigated.

Summer–autumn tea, a type of green tea, has experienced a sharp decline in economic value and considerable wastage due to its bitter taste and lower leaf quality than spring tea [14]. Notably, previous studies have indicated that the tea polysaccharide conjugates in summer–autumn tea are surprisingly higher than those in spring tea leaves [2,5,15]. Therefore, the extraction and utilization of summer and autumn tea polysaccharides can greatly increase the added value of summer and autumn tea. The polysaccharides extracted from fresh summer–autumn tea leaves using the hot water method had anti-diabetic functions [16]. However, there is a limited amount of research focused on the activity and structural characterization of summer–autumn tea polysaccharides. Thus, the purpose of this study is to isolate and purify the polysaccharide found in summer–autumn tea, and investigate the correlation between its structural characteristics and antioxidant activity. In this study, the polysaccharides extracted from summer–autumn tea were structurally characterized using a combination of high-performance liquid chromatography (HPLC), ultraviolet (UV) and Fourier-transform infrared (FT-IR) spectroscopy, gas chromatography–mass spectrometry (GC-MS), one-dimensional (1D) and two-dimensional (2D) nuclear magnetic resonance (NMR), and scanning electron microscopy (SEM). Furthermore, the antioxidant activities of the polysaccharides were also evaluated. Overall, this research aims to establish a theoretical framework for enhancing the value of summer–autumn tea and developing functional foods.

## 2. Materials and Methods

### 2.1. Materials

Summer–autumn tea (dried leaves) was provided by Jiangsu Tea Expo Park (Jurong, Jiangsu province, China) in August 2022. Standard monosaccharides of glucose (Glc), fructose (Fru), rhamnose (Rha), mannose (Man), arabinose (Ara), galactose (Gal), galacturonic acid (GalA), and papain were purchased from Aldrich (Sigma, Livonia, MI, USA). Sephacryl S-400 HR (26 mm × 1000 mm) was obtained from GE (Boston, MA, USA). Macroporous resin AB-8 and an anion-exchange chromatography column (DEAE seplife FF, 26 mm × 400 mm) were purchased from Lanxiao Technology New Materials Co., Ltd. (Xi’an, China). All other analytical reagents utilized were procured from Sinopharm Chemical Reagent Co., Ltd. (Shanghai, China).

### 2.2. Extraction of Crude Polysaccharides

The crude polysaccharides were extracted from the summer–autumn tea using hot water and then precipitated with ethanol. Initially, the dried tea leaves were crushed in a grinder and then passed through a 60-mesh sieve. In order to remove the alcohol-soluble substances in the tea leaves before the extraction of polysaccharides, ethanol (1:10, *w*/*v*) was used to dissolve the sample powder and the solution was stirred for 0.5 h at room temperature. The precipitate was obtained by centrifugation (6000× *g*) for 10 min. Subsequently, the precipitate and deionized water were mixed at a ratio of 1:10. After mixing, the mixture was placed in a water bath at 60 °C for 4 h. The supernatant extract was obtained through centrifugation at 6000× *g* for 10 min. The precipitate was extracted again according to the same procedure, and the supernatant was combined. The supernatant was concentrated to 1/10 (*v*/*v*) of its original volume by vacuum rotary evaporation, and then a four-fold volume of ethanol was added and reacted overnight to obtain the precipitate. Afterwards, the precipitate was redissolved in deionized water and dialyzed (Mw cut-off 3000 Da, Yuanye Biotechnology Co., Ltd., Shanghai, China) under running water for 3 days. Finally, the crude polysaccharide was obtained after the freeze-drying of the dialysate.

### 2.3. Purification of Crude Polysaccharide

Briefly, 20 g of crude polysaccharide was dissolved in 1000 mL deionized water along with 0.5–0.8‰ papain (*w*/*v*) and incubated at 25 °C overnight. Then, 250 mL of chloroform and butanol were added to the solution at a ratio of 4:1 (*v*/*v*); the mixture was shaken for 10 min, and the supernatant was obtained after centrifugation at 500 r/min for 10 min. Then, 300 mL of petroleum ether was added to the supernatant and mixed thoroughly, and then the lower aqueous phase was collected. Subsequently, macroporous resin AB-8 was added, mixed thoroughly, and adsorbed overnight. The collected supernatant was dialyzed and lyophilized to obtain polysaccharide (CPSA).

The CPSA polysaccharide (100 mg) was dissolved in 10.0 mL of distilled water and passed through a DEAE-cellulose column (26 mm × 40 cm) at a flow rate of 4.0 mL/min using varying concentrations of NaCl solution (0.1 M, 0.2 M, and 0.3 M). The fractions were collected in tubes containing 15 mL aliquots, and the sugar content was determined by employing the phenol sulfuric method after elution with distilled water [17]. The first fraction of D1 was then fractionated using a Sephacryl S-400 HR chromatography column (26 mm × 100 cm) with distilled water at a flow rate of 1.0 mL/min. The fractions were collected in 12 mL aliquots per tube, and the content was determined using the phenol–sulfuric acid method for the elution curve. The eluate from the same elution peak was combined, concentrated to 1/5 of the original volume by rotation under vacuum, and freeze-dried to obtain purified polysaccharides (marked as D1N1).

### 2.4. Ultraviolet and FT-IR Analysis

For the ultraviolet (UV) spectrum analysis, the D1N1 (0.5 mg/mL) solution was analyzed by a multimode reader (Multiskan GO, Thermo, Waltham, Massachusetts, USA) in the wavelength range of 200–800 nm. For the FT-IR spectrum analysis, 2 mg of D1N1 was mixed with 200 mg potassium bromide and pressed into 1 mm pieces with a tablet press. Then, the piece was scanned using a Nicolet iZ-10 Fourier-transform infrared spectrometer (Thermo, Massachusetts, USA) with a scanning range of 4000–450 cm^−1^.

### 2.5. Molecular Weight Analysis

The sample was dissolved in 0.1 M NaNO_3_ solution (containing 0.02% NaN_3_, *w*/*w*) until it reached a concentration of 1 mg/mL. After filtering through a 0.45 μm filter membrane, the filtrate was detected by gel permeation chromatography with a differential detector Optilab T-rEX (Wyatt technology, Goleta, CA, USA) and multiangle laser scattering analysis DAWN HELEOS Ⅱ (Wyatt technology, Goleta, CA, USA). Finally, the polysaccharide molecular weight (Mw) was determined with ASTRA 6.1 software.

### 2.6. Monosaccharide Analysis

The monosaccharide composition of D1N1 was determined following a previously reported method with certain modifications [18]. In detail, 5 mg of polysaccharide was hydrolyzed with 1 mL of 2 M trifluoroacetic acid (TFA) at 121 °C for 2 h, and the excess TFA was removed by blow-drying with nitrogen. The hydrolysate was washed with methanol, which was also blow-dried with nitrogen. This procedure was repeated three times to remove the excess TFA completely. Subsequently, the remaining substance was redissolved in deionized water and filtered through a 0.22 μm microporous filtering membrane for purification. The sample was subjected to analysis using high-performance anion-exchange chromatography (HPAEC) on a CarboPac PA-20 anion-exchange column (150 mm × 3 mm, Dionex, Sunnyvale, CA, USA), employing a pulsed amperometric detector (PAD; Dionex ICS 5000 + system). The flow rate was 0.5 mL/min and the injection volume was 5 μL. The mobile phase consisted of solvent system A (dd H_2_O), solvent system B (0.1 M NaOH), and solvent system C (0.1 M NaOH and 0.2 M NaAc). A gradient elution procedure was used for analysis, with solution A, B, and C being in a volume ratio of 95:5:0 at 0 min, 85:5:10 at 26 min, 85:5:10 at 42 min, 60:0:40 at 42.1 min, 60:40:0 at 52 min, and returning to a ratio of 95:5:0 at both the times of 52.1 min and 60 min. Data were obtained using the ICS 5000 + system (Thermo, Massachusetts, USA) and processed utilizing Chromeleon 7.2 CDS software (Thermo, Massachusetts, USA).

### 2.7. Methylation Analysis

The methylation method referred to the method reported by Ciucanu et al. [19]. The polysaccharide sample (2–3 mg) was dissolved in dimethyl sulfoxide (DMSO) and subsequently methylated using CH_3_I in a solution of DMSO/NaOH. After complete methylation, the permethylated products were subjected to hydrolysis using 2 M TFA at 121 °C for 1.5 h. Subsequently, reduction was carried out employing NaBD_4_, followed by acetylation acetic anhydride for 2.5 h at 100 °C. The acetates were dissolved in chloroform and subjected to GC-MS analysis using an Agilent 6890A-5975C system equipped with an Agilent BPX70 chromatographic column (30 m × 0.25 mm × 0.25 µm, SGE, Lingwood, Australia). Meanwhile, high-purity helium (split ratio 10:1) was employed as the carrier gas with a sample injection volume of 1 μL by Sanshu Biotech Co., Ltd. (Shanghai, China). Mass spectrometry analysis was initiated at a starting temperature of 140 °C and lasted for 2.0 min, followed by a gradual temperature ramp of 3 °C per minute until reaching 230 °C for an additional duration of 3 min. The scanning mode was SCAN with a range (*m*/*z*) from 50 to 350.

### 2.8. Nuclear Magnetic Resonance (NMR) Analysis

The sample was dissolved in 0.5 mL D_2_O to achieve a final concentration of 40 mg/mL. Subsequently, 1D-NMR and 2D-NMR (^1^H-NMR, ^13^C-NMR, COSY, NOESY, HMBC, and HSQC) were recorded at 25 °C using a Bruker AVANCE NEO 500M spectrometer system (Bruker, Rheinstetten, Germany) operating at 500 MHz by Sanshu Biotech. Co., Ltd. (Shanghai, China). The mixing time for NOESY is 0.6 s. D_2_O was used as the internal standard. 

### 2.9. Scanning Electronic Microscopy (SEM) Analysis

The molecular morphology of D1N1 was observed using the Zeiss Merlin Compact scanning electron microscope from Germany. The sample was placed on the substrate and coated with a thin gold layer. The images were subsequently observed at a voltage of 1.0 kV, utilizing a magnification of 5 k, in a high-vacuum environment by Sanshu Biotech Co., Ltd. (Shanghai, China).

### 2.10. Antioxidant Experiments

#### 2.10.1. 1,1-Diphenyl-2-picryl-hydrazyl (DPPH) Scavenging Experiment

This experiment referred to previous methods with some modifications [20]. First, DPPH was dissolved in 95% ethanol at a concentration of 8.62 × 10^−5^ mol/L. Then, 2.0 mL of polysaccharide solutions at varying concentrations (0.5, 1.0, 2.0, 3.0, and 4.0 mg/mL) were mixed thoroughly with 2.0 mL of DPPH solution. The vitamin C solution served as a positive control, and its concentration was consistent with the polysaccharide concentration. The mixture was incubated at room temperature for 30 min. In the control group, 95% ethanol was used instead of the polysaccharide solution. The absorbance of the mixture after incubation was measured at 517 nm. The DPPH scavenging activity was determined based on the following formula:

Scavenging activity (%) = [(A_0_ − A_1_)/A_0_] × 100

where A_0_ and A_1_ represent the absorbance value of the DPPH solution and the polysaccharide sample mixed with the DPPH solution at 517 nm, respectively. 

#### 2.10.2. •OH Scavenging Experiment

The method utilized in the study adhered to the procedure outlined by Ji et al. [21]. Briefly, 2 mL of polysaccharide solution (the concentration is consistent with Section 2.10.1) and vitamin C sample were added into test tubes separately. Then, 2 mL of salicylic acid solution (6 mmol/L), 2 mL of FeSO_4_ solution (6 mmol/L), 2 mL of distilled water, and 6 mmol/L H_2_O_2_ were added into the test tube. All mixtures were thoroughly mixed and incubated at 37 °C for 30 min. In the blank group, the tea polysaccharide solution was substituted with distilled water. The absorbance was measured at a wavelength of 510 nm. The •OH scavenging activity was determined based on the following formula:

Scavenging activity (%) = [(A_0_ − A_s_)/A_0_] × 100

where A_0_ and A_s_ represent the absorbance value of the blank group and sample group at 510 nm, respectively.

#### 2.10.3. ABTS^+^ Scavenging Experiment

This experiment was based on Abuduwaili’s method with slight modifications [22]. Here, 2.9 mL of ABTS^+^ solution (2.45 mmol/L) was added to 0.1 mL of polysaccharide solution (the concentration is consistent with Section 2.10.1) and incubated at 30 °C for 10 min. In the blank group, distilled water was used instead of the tea polysaccharide solution. The absorbance of the mixture was measured at a wavelength of 734 nm. The ABTS^+^ scavenging activity was determined based on the following formula:

Scavenging activity (%) = [(A_0_ − A_m_)/A_0_] × 100

where A_0_ and A_m_ represent the absorbance value of the blank group and sample group at 734 nm, respectively.

### 2.11. Statistical Analysis

All experiments were carried out in triplicate and the results were reported as means ± standard deviation (SD). One-way ANOVA and Duncan’s test were employed to assess the statistical significance of multiple groups using SPSS statistical software version 20.0. Origin 9 was employed to conduct a comparison of categorical variables and generate visual representations. Different letters indicated a statistically significant difference (*p* < 0.05).

## 3. Results and Discussion

### 3.1. Extraction and Purification of Polysaccharide from Summer–Autumn Tea

**Figure 1:**
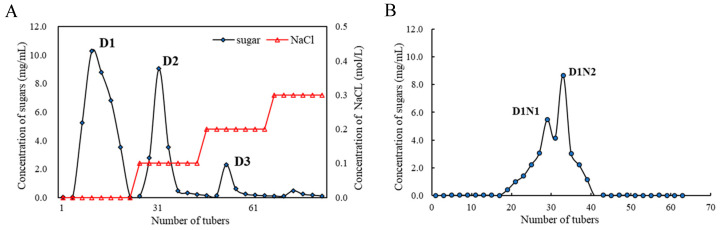
Elution curves of crude polysaccharide from summer–autumn tea on DEAE-52 anion-exchange column (**A**). Elution curves of D1 on Sephacryl S-400 HR chromatography column (**B**).

The yield of CPSA in the summer–autumn tea was 9.3% after hot water extraction and ethanol precipitation. After removing impurities with macroporous resin, the recovery rate of the CPSA was 18.5%. Meanwhile, an additional measure was implemented to isolate the CPSA by utilizing the DEAE-52 cellulose anion-exchange column. The results revealed that there were three fractions in the CPSA solution (Figure 1A), which were labeled as D1, D2, and D3 according to the order of peak emergence. The proportions of D1, D2, and D3 were 72.85%, 22.24%, and 4.91%, respectively. Since the proportion of the D1 component was the highest, a Sephacryl S-400 HR chromatography column was used to further purify the D1 fraction. As exhibited in Figure 1B, the D1 fraction contained two components named D1N1 and D1N2. The proportions of D1N1 and D1N2 were 45.88% and 49.63%, respectively. The monosaccharide compositions of D1N1 and D1N2 were different, and should be different components. And the monosaccharide composition of D1N1 was more abundant. In subsequent experiments, D1N1 was selected for further structural and antioxidant activity analysis.

### 3.2. UV and FT-IR Analysis of D1N1

**Figure 2:**
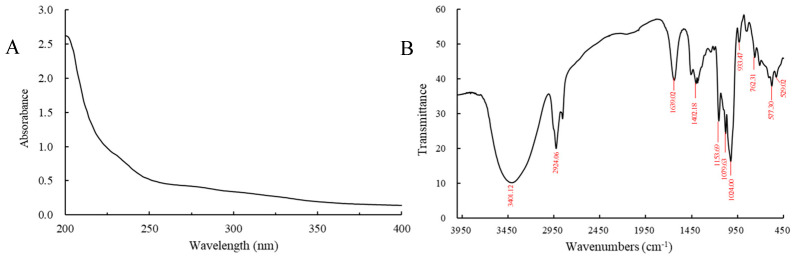
UV (**A**) and FT-IR (**B**) spectra of D1N1.

As shown in Figure 2A, the sample displayed no evident absorption in the wavelength range of 200–400 nm, indicating that there were no impurities such as pigments, proteins, or nucleic acids in the sample. The FT-IR spectrum of the D1N1 is exhibited in Figure 2B. The absorption band observed in the range of 3200–3600 cm^−1^ corresponds to the stretching vibration of O-H groups, which serves as a distinctive characteristic peak for polysaccharide chains [23]. Additionally, the absorption peak observed at 2924.06 cm^−1^ can be attributed to the C-H stretching vibration [24], while the absorption peak observed at 1639.02 cm^−1^ belongs to the C=O stretching vibration [25]. The absorption bands observed at 1153 cm^−1^ and 1079 cm^−1^ belong to C-O-C and C-OH stretching vibrations, respectively [26,27]. The band around 930 cm^−1^ was caused by the asymmetric ring vibration of D-pyranosides [27]. Moreover, the absorption peaks observed at 700–900 cm^−1^ determine the presence of the skeleton structure of the pyranose ring [23], and the vibration around 765 cm^−1^ refers to α-glycoside [28]. Therefore, these results indicated that D1N1 had the structural characteristics of the polysaccharide.

### 3.3. Molecular Weight (Mw) of D1N1

**Figure 3:**
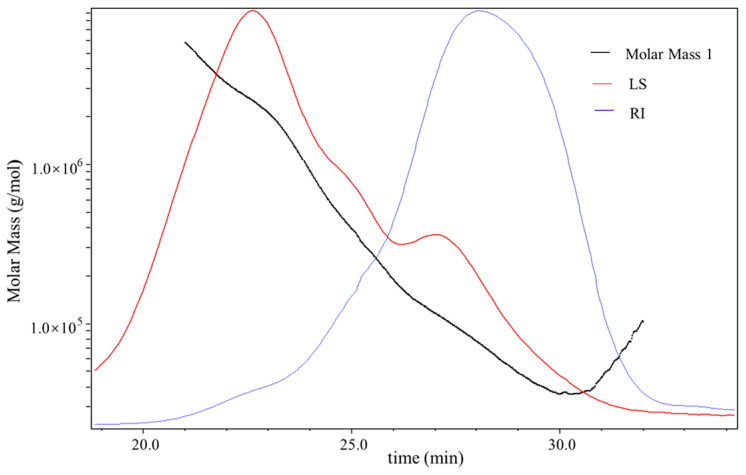
Chromatograms of the molar mass distribution of D1N1. The variation tendencies of signals of the multi-angle laser scattering (LS), refractive index (RI), and fitted molar mass of D1N1. The red line indicates the variation tendency in the LS of D1N1 with retention time, and the blue line represents the signals collected by the refractive index (RI) detectors; the dark line is the varying tendency of the molar mass fitted by the LS and RI signal of the D1N1 following the retention time.

The molecular mass distribution of D1N1 is presented in Figure 3. The RI signal for D1N1 had only one peak, indicating the homogeneity of D1N1. The black line was the molecular weight (Mw) fitted from the blue and green signals. Mw is calculated from the Mark–Houwink equation. The absolute Mw of D1N1 was determined to be 224.71 kDa by processing the data using the software ASTRA 6.1. Meanwhile, the polydispersity (Mw/Mn) of D1N1 was 3.13, indicating that the molar mass distribution range was relatively narrow. It was reported that the Mw of polysaccharides from diverse tea sources ranges from 0.472 to 3140 kDa [7]. Li et al. employed high-performance gel permeation chromatography (HPGPC) to determine the molecular weight (Mw) of green tea polysaccharides (GTPSs), revealing a value of 96.9 kDa [29]. Additionally, the Mw of TP-12, a polysaccharide fraction from yellow tea, was determined to be 224.7 KDa using HPSEC-RID analysis [6]. Jia et al. employed gel permeation chromatography (GPC) to determine the molecular weights (Mws) of two constituents, HMP-1 and HMP-2, in the mature leaves of Hawk tea, yielding estimated values of 133 kDa and 100 kDa, respectively [30]. These results indicated that the Mw of polysaccharides was related to the type of tea leaves.

### 3.4. Monosaccharide Analysis of D1N1

In order to better understand the structure and activity of D1N1, the HPAEC-PAD method was applied to determine the monosaccharide composition of D1N1. As listed in Table 1, the composition of D1N1 included four monosaccharides, namely, arabinose, galactose, glucose, and rhamnose. Glucose was the main constituent in the structure of D1N1, with a molar ratio of 95.3%. The proportion of the other three monosaccharides was relatively low, accounting for about 5% in total. Yin et al. reported a neutral polysaccharide from green tea, in which the highest proportion of monosaccharides was glucose, accounting for 90% [31]. This finding was similar to the results of our research. However, Guo et al. reported that the monosaccharides of the polysaccharides (BPT-1, BPT-2, and BPT-3) in green tea powder were all composed of arabinose, rhamnose, glucose, and galactose, but galactose and arabinose were predominantly present and accounted for about 70% of the total sugars [32]. Thus, the composition of monosaccharides in polysaccharides derived from various tea species may exhibit discrepancies [7].

### 3.5. Linkage Analysis of D1N1

To further identify the structural information of D1N1, the glycosidic bond types were analyzed by methylation and GC–MS methods. The linkage patterns of D1N1 were determined by comparing the retention time and ion fragment characteristics of methylated polysaccharides with the database. The results showed that the derivatives of 1,5-di-O-acetyl-2,3,4,6-tetra-O-methyl glucitol, 1,4,5-tri-O-acetyl-2,3,6-tri-O-methyl glucitol, 1,3,4,5-tetra-O-acetyl-2,6-di-O-methyl galactitol and 1,4,5,6-tetra-O-acetyl-2,3-tri-O-methyl glucitol were detected in D1N1. These results also indicated that D1N1 possessed the glycosidic bond of terminal Glcp (1→, →4)-Glc*p* (1→, →3,4)-Gal*p* (1→ and →4,6)-Glc*p* (1→. The linkage patterns and their molar ratios are summarized in Table 2. It should be noted that the monosaccharide composition contained rhamnose and arabinose, which were absent from the methylation results, possibly because the content of these two sugars was too low to detect the derivatives. Furthermore, similar results have also been reported in other literatures [33,34].

### 3.6. NMR Analysis of D1N1

**Figure 4:**
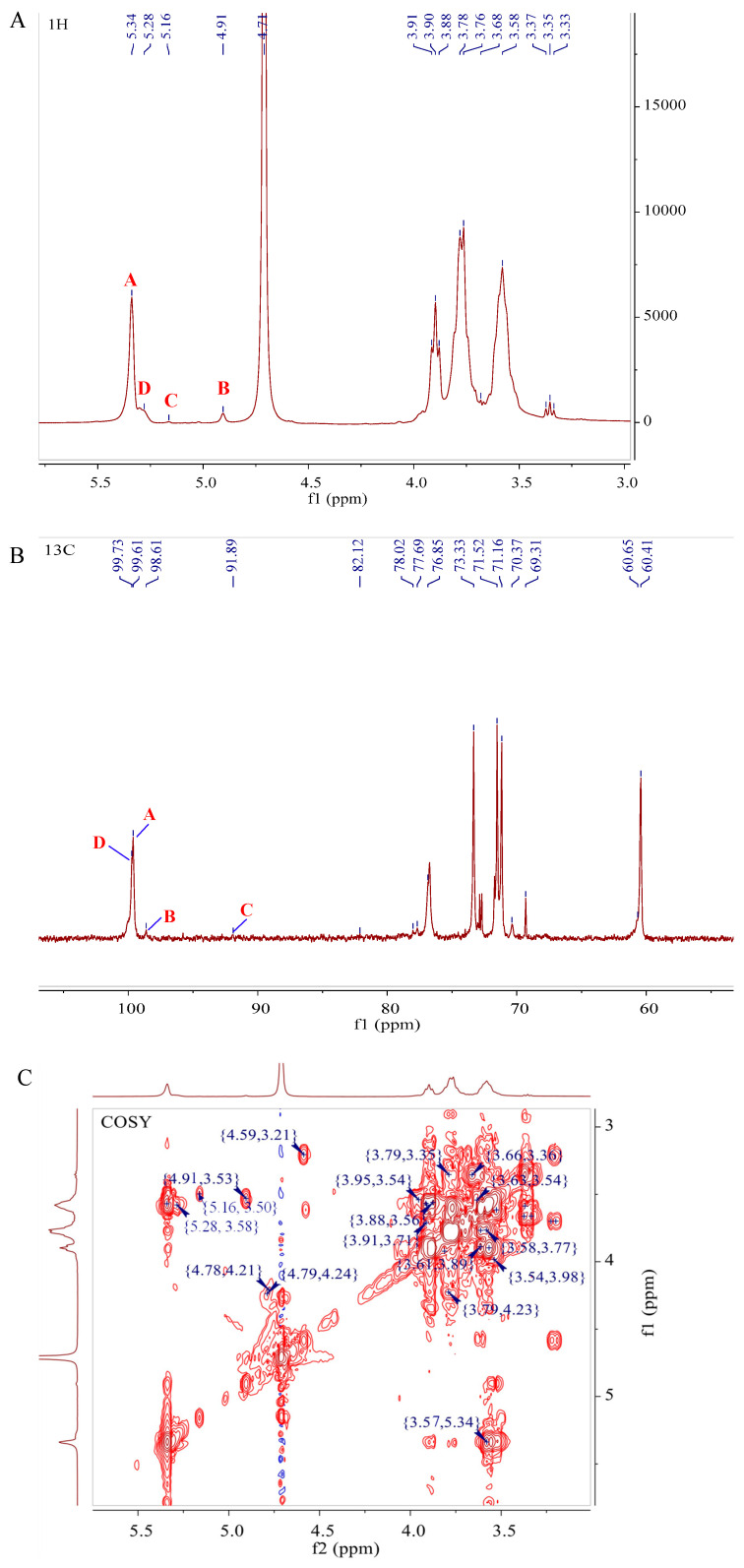

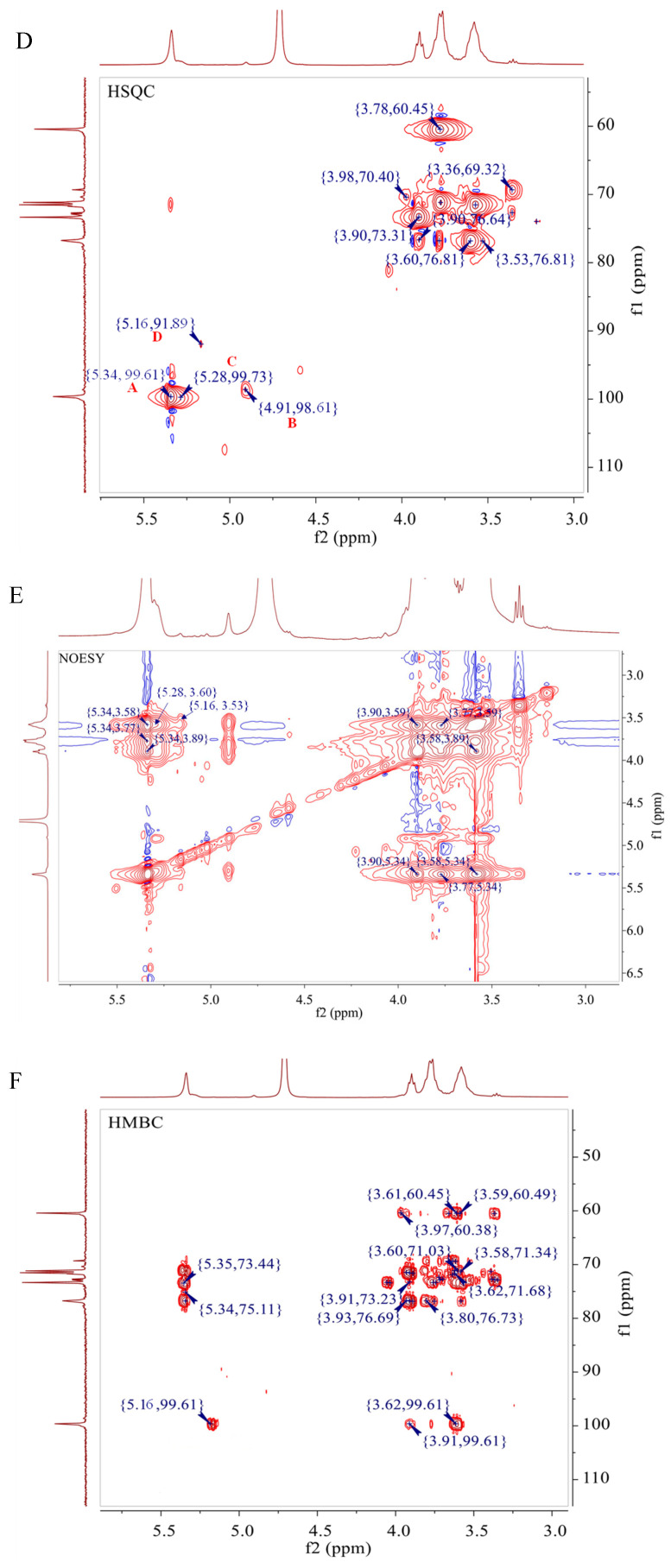
NMR spectra and proposed structure of D1N1. ^1^H NMR spectrum (**A**), ^13^C NMR spectrum (**B**), ^1^H-^1^H COSY spectrum (**C**), ^1^H-^13^C HSQC spectrum (**D**), NOESY spectrum (**E**), and HMBC spectrum (**F**).

NMR spectroscopy is the remarkably efficient and noninvasive technique for elucidating the structure of polysaccharides. It possesses the capability to provide comprehensive insights into the structure of polysaccharides without destroying them, including the identification of monosaccharide composition, the elucidation of α- or β-anomeric configurations, and the connection sequence of glycosidic bonds [35]. To further enhance the understanding of D1N1’s structure, we performed NMR spectroscopy in both 1D and 2D formats. In the ^1^H NMR spectra (Figure 4A), the signal of polysaccharides appeared at 3–6 ppm [36]. Generally, the anomeric hydrogen signals of the β-glycosidic bond configuration are mainly distributed at δ 4.3–4.8 ppm, and those of the α-glycosidic bond configuration are mainly distributed at δ 4.8–5.8 ppm [36,37]. Four signal peaks were identified in the anomeric signal area of D1N1, suggesting that D1N1 contained three kinds of sugar residues. The chemical shifts were δ 5.34, 5.28, 5.16, and 4.91 ppm and the residues were labeled as A, D, C, and B, respectively. 

Compared with ^1^H NMR, the distribution of chemical shift signals in ^13^C NMR for polysaccharides is more extensive (Figure 4B). The ^13^C NMR spectra of polysaccharides typically exhibit predominant anomeric carbon signals in the range of 90–110 ppm, while chemical shifts for C2-C6 are commonly observed between 60 and 80 ppm. In addition, the presence of four anomeric carbon signals was detected at δ 99.73 ppm, δ 99.61 ppm, δ 98.61 ppm, and δ 91.89 ppm. Combined with the ^13^C NMR spectrum and cross peaks of the HSQC spectrum (Figure 4D) in the anomeric region, the anomeric signals of A, B, C, and D were determined to be δ 5.34/99.61, δ 4.91/98.61, 5.16/91.89, and δ 5.28/99.73, respectively. Then, based on the ^1^H-^1^H COSY spectrum (Figure 4C), a potential correlation was observed between the H-1 of residue A at 5.34 ppm and H-2 at 3.57 ppm. Similarly, the analysis of the spectrum revealed that H-3 to H-6 was identified at 3.98, 3.6, 3.77, and 3.62 (3.78) ppm. Based on the HSQC signals, the chemical shifts of C2–C6 on residue A were δ 71.53 ppm, δ 70.4 ppm, δ 76.81 ppm, δ 71.18 ppm, and δ 60.45 ppm, respectively. It is worth noting that the chemical shift of C-4 of residue A was approximately 77 ppm, indicating that substitution occurred at the C-4 position [38]. The same analytical method was used to assign the chemical shifts of residue B, residue C, and residue D. In conjunction with the results of methylation analysis, monosaccharide composition, NMR results, and literature reports [39,40,41], it could be inferred that residues A, B, C, and D correspond to →4)-α-d-Glc*p* (1→, α-d-Glc*p*-(1→, →3,4)-α-d-Gal*p*-(1→ and →4,6)-α-d-Glc*p*-(1→. The chemical shifts of proton and carbon are summarized in Table 3.

**Figure 5:**
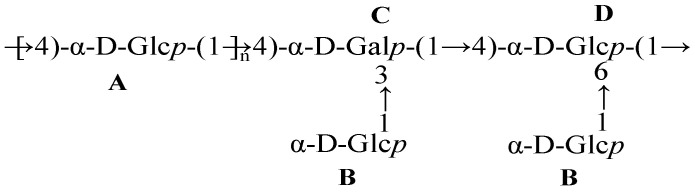
Proposed structure of repeating units of D1N1.

Based on the heteronuclear multiple bond correlation (HMBC, Figure 4F) and nuclear Overhauser effect spectroscopy (NOESY, Figure 4E) spectra, the linkage of glycoside residues in D1N1 was speculated. In Figure 4F, the cross-peak signals between H-1 (δ 5.34, residue A)/C-4 (δ 76.81, residue A), H-1 (δ 5.34, residue A)/C-4(δ 76.64, residue C), C-1 (δ 99.61, residue A)/H-4 (δ 3.6, residue A), C-1 (δ 99.61, residue A)/H-4 (δ 3.9, residue C) and C-1 (δ 99.73, residue D)/H-4 (δ 3.9, residue A) could be obtained from the HMBC spectrum of D1N1. In the NOESY spectrum shown in Figure 4E, a correlation signal was observed between H-1 of residue A and H-4 of residue A, suggesting the existence of A-A links. The identification of a correlation signal between H-1 of residue A and H-4 of residue C provides additional evidence for the existence of links. In addition, H-1 of residue B had cross-peaks with H-3 of residues C and H-6 of residue D, respectively. The cross-peak between H-1 of residue C and H-4 of residue D was also observed. Furthermore, H-1 of residue D had cross-peaks with H-4 of residue A. From combining all of the results, the possible linkage sequence of D1N1 was deduced to be a heteropolysaccharide, with a backbone structure of →4)-α-d-Glc*p* (1→, →3,4)-α-d-Gal*p*-(1→ and →4,6)-α-d-Glc*p* (1→ residues and side chains consisting of α-d-Glc*p*-(1→. The proposed structure of repeating units of D1N1 was determined (Figure 5). The results of methylation showed that the ratio of →4)-α-d-Glcp-(1→, →3,4)-α-d-Gal*p*-(1→) and →4,6)-α-d-Glc*p* (1→ was approximately 36:1:1.5, with a low proportion of →3,4)-α-d-Gal*p*-(1→). Similarly, the ^1^H NMR spectrum also revealed that the peak of→3,4)-α-d-Gal*p*-(1→) was too small for its area to be calculated. Therefore, it was estimated that the value of the repeating unit number (n value in Figure 5) was approximately 35 based on the methylation results and the final sugar chain structure.

### 3.7. SEM Analysis of D1N1

**Figure 6:**
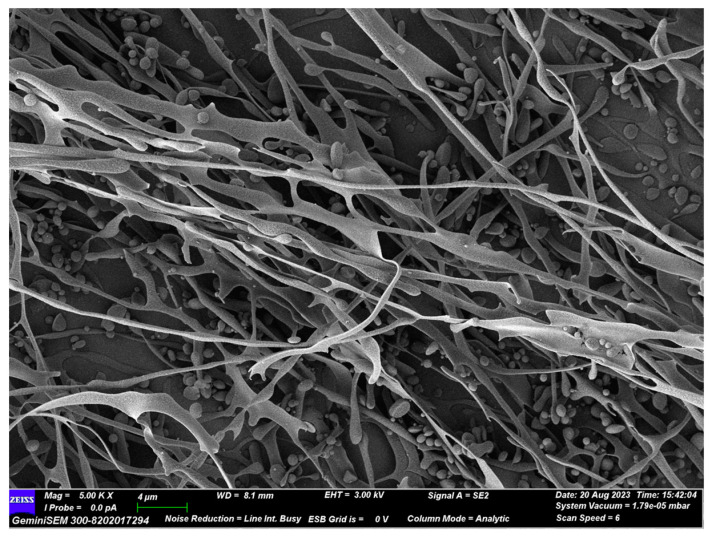
SEM image of D1N1 at 5000× magnification.

SEM, as a microscopic molecular morphology observation technique, is commonly used to characterize the surface morphology of polysaccharides [33]. The SEM morphology of D1N1 is displayed in Figure 6; it can be observed that the polysaccharide sample showed irregular bands with a localized spherical granular structure at 5 k magnification. Liu et al. reported that, according to SEM analysis, the water-soluble polysaccharide extracted from dark brick tea (DTP-1) exhibited irregular flakes [8]. The surface morphology of Liupao tea showed granular agglomerates stacked together at 5 k magnification, as reported by Wei et al. [42]. Moreover, Chen et al. found a green polysaccharide (gTPC-T and gTPC-1) in a lamellar morphology, which consisted of a series of fragments [43]. In contrast, the morphology of gTPC-2, which has a relatively small molecular weight, is composed of agglomerates of fine powdery particles. These phenomena indicated that the morphological characteristics of tea polysaccharides may be related to their molecular weight, type of tea, extraction method, etc.

### 3.8. Antioxidant Activity of D1N1 In Vitro

**Figure 7:**
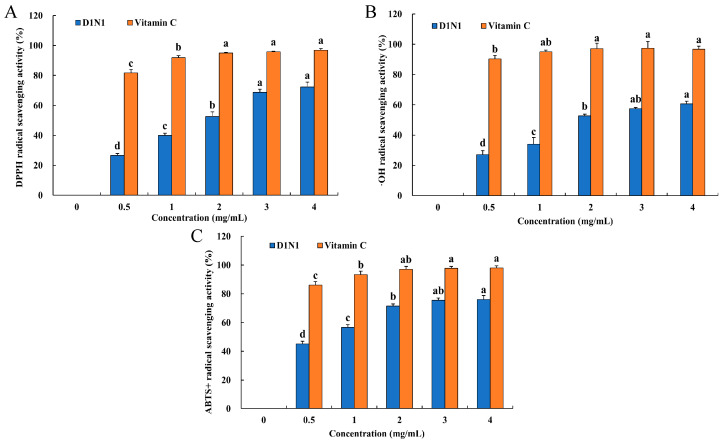
Evaluation of the antioxidant activity of D1N1. The DPPH radical (**A**), OH radical (**B**), and ABTS+ radical (**C**) scavenging activity of D1N1. The lowercase letters (a–d) on the bars represent a statistically significant difference (*p* < 0.05) among the various concentrations.

Many recent studies have revealed that tea polysaccharides have significant antioxidant activity [32,44,45]. The antioxidant activity of D1N1 in vitro was investigated by DPPH, •OH, and ABTS^+^ scavenging assay (Figure 7). As shown in Figure 7A, D1N1 showed obvious concentration-dependent DPPH scavenging activity. When the concentration of polysaccharide reached 4.0 mg/mL, the scavenging rate of DPPH was up to 72.13%, which was significantly higher than the polysaccharide concentration range of 0.5–2.0 mg/mL (*p* < 0.05). These results indicated that D1N1 possessed good antioxidant activity.

In addition, OH radicals are widely used to estimate the antioxidant ability of compounds. As illustrated in Figure 7B, when the polysaccharide concentration was low, the scavenging activity of •OH was weak. When the concentration of polysaccharide was below 2.0 mg/mL, there was a significant increase in the scavenging capacity of D1N1 (*p* < 0.05). When the concentration of polysaccharide exceeded 2.0 mg/mL, the scavenging ability of D1N1 continued to exhibit an upward trend, but was not statistically significant (*p* < 0.05). These results demonstrated that the polysaccharide solution with higher concentrations exhibited a better scavenging activity.

ABTS^+^ is another free radical in the antioxidant evaluation system of compounds. In Figure 7C, D1N1 also exhibited excellent ABTS+ radical scavenging activity in a concentration-dependent manner. When the concentration of D1N1 increased from 0.5 to 4.0 mg/mL, the scavenging activity of ABTS^+^ free radical was effectively enhanced. D1N1 possessed the highest ABTS^+^ radical scavenging activity (73.2%) at a concentration of 4.0 mg/mL, which was significantly higher than the scavenging activity of D1N1 at 0.5–2.0 mg/mL (*p* < 0.05). Moreover, this result was higher than the previous report [42]. However, the scavenging ability of D1N1 was lower compared with that of vitamin C. The results indicated that D1N1 had comprehensive antioxidant activity.

The activity of polysaccharides has been reported to be related to structural characteristics, such as monosaccharide composition, molecular weight, and glycosidic bond type [46]. According to a previous report, Mw is a critical factor for the antitumor activity of polysaccharides, and the intermediate Mw facilitates the polysaccharides to exert their antioxidant activity [47,48]. In our present study, the Mw of D1N1 was in good agreement with the findings of the existing literature. In addition, monosaccharide composition may be related to the antioxidant activity of polysaccharides. Previous studies have indicated that the antioxidant activity of polysaccharides is associated with the ratio of different monosaccharide components, with rhamnose and galactose being important factors [13]. In terms of monosaccharide composition, D1N1 contained rhamnose and galactose. Despite its relatively low content, D1N1 still exhibited good antioxidant capacity. Besides this, the extraction techniques employed for TPS can exert an influence on its antioxidant activity. For instance, Wang et al. conducted a comparative analysis of four different methods (freeze-drying, vacuum-drying, spray-drying, and microwave-vacuum-drying) to obtain TPSs and assessed their respective antioxidant activities [49]. The TPS obtained through the freeze-drying process (TPS-F, 42.71%) demonstrated enhanced efficacy in scavenging metal-chelated and superoxide radicals compared to others. The reason may be that the rough and porous surface of TPS-F is conducive to increasing the solubility of polysaccharides and enhancing their antioxidant activity. Similarly, in this study, D1N1 was obtained by freeze-drying. The SEM image (Figure 6) also showed that D1N1 appeared in a loose band shape, which could potentially contribute to the robust antioxidant capacity exhibited by D1N1. As mentioned above, the activity of polysaccharides is influenced by multiple factors rather than being solely determined by a single factor. Therefore, the antioxidant mechanism of polysaccharides is relatively complex.

## 4. Conclusions

In this study, we presented the structural properties and antioxidant potential of a polysaccharide (D1N1) extracted from summer–autumn tea. D1N1 was found to be a heteropolysaccharide, which consists of arabinose, rhamnose, glucose, and galactose, with glucose accounting for the largest proportion. Meanwhile, the Mw of D1N1 was 224.8 kDa, and the detailed structure of D1N1 was elucidated in this study. Furthermore, D1N1 exhibited excellent antioxidant bioactivity against the DPPH, •OH, and ABTS^+^ radicals. In summary, this study can promote the utilization of summer–autumn tea and provide a theoretical basis for subsequent research on polysaccharides from summer–autumn tea.

## Figures and Tables

**Table 1:** Monosaccharide composition of D1N1.

Sample	DIN1			
Monosaccharide composition	Rhamnose	Arabinose	Galactose	Glucose
Molar ratio (%)	0.44	2.43	1.83	95.30

**Table 2:** Linkage analysis data of D1N1.

Sample	Type of Linkage	Methylated Sugars	Retention Time	Mass Fragments	Relative Molar Ratio (%)
D1N1	t-Glc(*p*)	1,5-di-O-acetyl-2,3,4,6-tetra-O-methyl glucitol	8.994	55.1, 71.1, 87.1, 102.1, 129.1, 145.1, 162.1, 205.1, 239.3	11.83
	4-Glc(*p*)	1,4,5-tri-O-acetyl-2,3,6-tri-O-methyl glucitol	14.244	71, 87, 102, 118, 129, 142, 162, 233	82.36
	3,4-Gal(*p*)	1,3,4,5-tetra-O-acetyl-2,6-di-O-methyl galactitol	16.308	59, 87, 99, 118, 129, 143, 185	2.31
	4,6-Glc(*p*)	1,4,5,6-tetra-O-acetyl-2,3-tri-O-methyl glucitol	18.414	59, 74, 85, 102, 118, 127, 142, 162, 201, 261	3.50

**Table 3:** Chemical shifts (ppm) of ^1^H and ^13^C signals for the D1N1 recorded in D_2_O at 313 K.

Code	Glycosyl Residues	Chemical Shifts (ppm)					
		H1/C1	H2/C2	H3/C3	H4/C4	H5/C5	H6/C6
A	→4)-α-d-Glc*p*-(1→	5.34	3.57	3.98	3.60	3.77	3.62, 3.78
		99.61	71.53	70.4	76.81	71.18	60.45
B	α-d-Glc*p*-(1→	4.91	3.53	3.90	3.36	3.57	3.62, 3.92
		98.61	71.40	73.31	69.31	71.53	60.79
C	→3,4)-α-d-Gal*p*-(1→	5.16	3.50	3.76	3.90	3.49	3.65, 3.90
		91.89	70.00	76.78	76.64	70.30	61.30
D	→4,6)-α- d -Glc*p*-(1→	5.28	3.58	3.87	3.53	3.90	3.87
		99.73	72.52	71.33	76.81	71.82	69.80

## Data Availability

The original contributions presented in the study are included in the article, further inquiries can be directed to the corresponding author.
